# Chronic pain enhances excitability of corticotropin-releasing factor-expressing neurons in the oval part of the bed nucleus of the stria terminalis

**DOI:** 10.1186/s13041-024-01094-6

**Published:** 2024-05-03

**Authors:** Ryoko Uchida, Yasutaka Mukai, Taiju Amano, Kenji Sakimura, Keiichi Itoi, Akihiro Yamanaka, Masabumi Minami

**Affiliations:** 1 Department of Pharmacology, Graduate School of Pharmaceutical Sciences, Hokkaido University, Sapporo, 060-0812 Japan; 2 Department of Neuroscience II, Research Institute of Environmental Medicine, Nagoya University, Nagoya, 464-8601 Aichi Japan; 3 Department of Animal Model Development, Brain Research Institute, Niigata University, Niigata, 951-8585 Japan; 4 Department of Nursing, Tohoku Fukushi University, Sendai, 981-8522 Japan; 5 Department of Cellular Pharmacology, Graduate School of Medicine, Hokkaido University, Sapporo, 060-8638 Japan; 6 Chinese Institute for Brain Research, Beijing, 102206 China; 7 Division of Brain Sciences, Institute for Advanced Medical Research, Keio University School of Medicine, Shinjuku, Tokyo, 160-8582 Japan; 8 National Institute for Physiological Sciences, National Institutes of Natural Sciences, Okazaki, 444-8585 Aichi Japan; 9 Postdoctoral Research Fellow, Japan Society for the Promotion of Science, Tokyo, 102-0083 Japan

**Keywords:** Bed nucleus of the stria terminalis, Chronic pain, Corticotropin-releasing factor, Neuronal excitability, Negative emotion

## Abstract

We previously reported that enhanced corticotropin-releasing factor (CRF) signaling in the bed nucleus of the stria terminalis (BNST) caused the aversive responses during acute pain and suppressed the brain reward system during chronic pain. However, it remains to be examined whether chronic pain alters the excitability of CRF neurons in the BNST. In this study we investigated the chronic pain-induced changes in excitability of CRF-expressing neurons in the oval part of the BNST (ovBNST^CRF^ neurons) by whole-cell patch-clamp electrophysiology. CRF-Cre; Ai14 mice were used to visualize CRF neurons by tdTomato. Electrophysiological recordings from brain slices prepared from a mouse model of neuropathic pain revealed that rheobase and firing threshold were significantly decreased in the chronic pain group compared with the sham-operated control group. Firing rate of the chronic pain group was higher than that of the control group. These data indicate that chronic pain elevated neuronal excitability of ovBNST^CRF^ neurons.

**Supplementary Information:**

The online version contains supplementary material available at 10.1186/s13041-024-01094-6.

## Introduction

Pain-induced aversive responses are important for the physiological role of pain as a biological warning system. However, chronic pain induces maladaptive emotional states, which often lead to psychiatric disorders, such as depression and anxiety disorders. Therefore, it is important to elucidate the neural mechanisms of chronic pain-induced maladaptive emotional states. We have reported that enhanced release of corticotropin-releasing factor (CRF) in the anterolateral part of bed nucleus of the stria terminalis (BNST) is involved in acute pain-induced aversive responses [1], and that sustained enhancement of CRF signaling in the anterolateral BNST during chronic pain suppresses the brain reward system, which may lead to depression-like states [2]. However, it remains to be examined whether chronic pain alters the excitability of CRF neurons in the BNST. Thus, in this study we investigated the chronic pain-induced changes in excitability of CRF-expressing neurons in the oval part of the BNST (ovBNST^CRF^ neurons), where CRF neurons are densely located, by whole-cell patch-clamp electrophysiology using brain slices prepared from a mouse model of neuropathic pain.

## Materials and methods

**Fig. 1:**
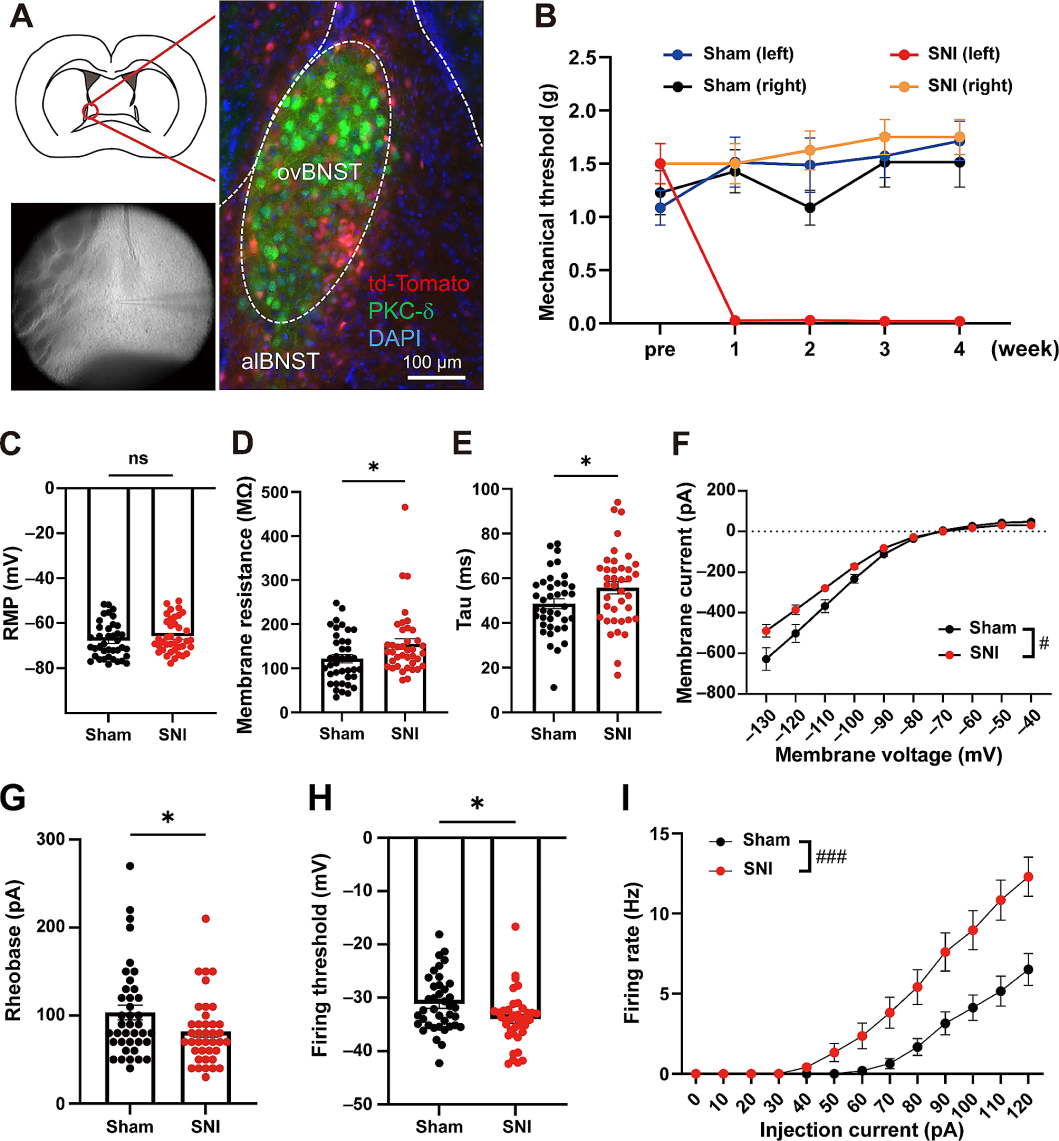
Chronic pain enhances excitability of CRF neurons in the ovBNST. A, Electrophysiological recordings from ovBNST^CRF^ neurons labeled by tdTomato. Immunohistological analysis using an antibody for PKCδ, which specifically localize in the ovBNST, was conducted to confirm the localization of tdTomato-positive CRF-expressing neurons in the ovBNST. B, Time courses of pain thresholds in the SNI (*n* = 8) and sham (*n* = 7) groups. C-F, Intrinsic electrophysiological properties of ovBNST^CRF^ neurons: RMP (C; sham: − 67.77 ± 1.26 mV vs. SNI: − 65.80 ± 1.21 mV, *t*_76_ = 1.129, *P* = 0.2625), membrane resistance (D; sham: 121.7 ± 9.1 MΩ vs. SNI: 155.5 ± 11.8 MΩ, *t*_76_ = 2.271, *P* = 0.026), tau (E; sham: 48.60 ± 2.18 ms vs. SNI: 55.76 ± 2.76 ms, *t*_76_ = 2.037, *P* = 0.0451), and I-V curve (F; interaction, *F*_(9, 684)_ = 5.441, *P* < 0.0001, group: *F*_(1, 76)_ = 4.884, *P* = 0.0301, membrane voltage: *F*_(9, 684)_ = 309.3, *P* < 0.0001). G-I, Neuronal excitability of ovBNST^CRF^ neurons: rheobase (G; sham: 103.6 ± 8.4 pA vs. SNI: 81.8 ± 6.0 pA, *t*_76_ = 2.105, *P* = 0.0386), firing threshold (I; sham: − 31.16 ± 0.84 mV vs. SNI: − 33.97 ± 0.79 mV, *t*_76_ = 2.443, *P* = 0.0169), and firing rate (J; interaction: *F*_(12, 744)_ = 9.411, *P* < 0.0001, group: *F*_(1, 62)_ = 12.36, *P* = 0.0008, injection current: *F*_(12, 744)_ = 82.09, *P* < 0.0001). Data are expressed as means ± standard error of the mean. ^ns^*P* > 0.05, ^*^P < 0.05 (unpaired *t*-test), ^#^*P* < 0.05, ^###^*P* < 0.001 (two-way repeated measures ANOVA)

CRF-Cre [3]; Ai14 mice on C57BL/6J background were used to visualize ovBNST^CRF^ neurons. In this study, we followed the Allen Mouse Brain Atlas for the anatomical terminology of the subnuclei within the BNST [4]. Immunohistological analysis using an antibody for PKCδ, which specifically localize in the oval part within the BNST (ovBNST) [5], was conducted to confirm the localization of CRF-expressing neurons in the ovBNST (Fig. 1A). A mouse model of neuropathic pain (spared nerve injury model, SNI) was prepared by ligating then cutting the tibial and common peroneal nerves on the left side [6] under anesthesia with isoflurane (induction, 3.0%; maintenance, 2.0%). The von Frey test was performed 1 day before and 1, 2, 3, and 4 weeks after the surgery to confirm the induction of chronic pain. (Fig. 1B). Four to five weeks after the surgery, mice were sacrificed and the brain slices including the BNST were prepared for whole-cell patch-clamp recordings from ovBNST^CRF^ neurons. Resting membrane potential, membrane resistance, tau, and rheobase were measured. The action potential threshold was defined as the membrane potential at which the derivative of the voltage (dV/dt) exceeded 10 mV/ms. The detailed materials and methods were described in the additional information 1. Data indicate means ± SEM. Statistical analyses were conducted using GraphPad Prism (GraphPad Software Inc., La Jolla, CA, USA). Two-tailed unpaired *t* test and two-way repeated measures ANOVA were used to analyze the data as shown in the figure legend. Differences with *P* < 0.05 were considered significant.

## Results

Electrophysiological recordings were carried out from ovBNST^CRF^ neurons (sham: *n* = 39 cells from 7 mice, SNI: *n* = 39 cells from 8 mice) labeled by tdTomato. Although resting membrane potential (RMP) was indistinguishable between the SNI and sham groups (Fig. 1C), membrane resistance (Fig. 1D) and tau (Fig. 1E) were significantly increased in the SNI group. In the I-V curve, inward rectifying current was observed at higher membrane potentials in both the SNI and sham groups. Negative membrane current was smaller in the SNI group at lower membrane potentials (Fig. 1F). These data suggest that chronic pain altered intrinsic electrophysiological properties of ovBNST^CRF^ neurons. Next the neuronal excitability was examined in SNI and sham groups. Rheobase (Fig. 1G) and firing threshold (Fig. 1H) were significantly lower in the SNI group compared with the sham group. The number of action potentials evoked by + 10 pA step current (500-ms duration) across the range of 0-120 pA was measured in 31 and 33 cells of the sham and SNI groups, respectively. The firing rate of the SNI group was higher than that of the sham group (Fig. 1I). These data indicate that chronic pain elevated neuronal excitability of ovBNST^CRF^ neurons.

## Discussion

We previously reported that sustained enhancement of CRF signaling within the BNST during chronic pain suppresses the dopaminergic neurons in the ventral tegmental area [2]. However, it remains to be examined whether chronic pain alters the neuronal excitability of CRF neurons in the BNST. In this study, we utilized CRF-Cre; Ai14 mice to visualize CRF-expressing neurons in the brain slices prepared from the mouse model of neuropathic pain and examined chronic pain-induced changes in excitability of ovBNST^CRF^ neurons. The results showed that chronic pain elevated neuronal excitability of ovBNST^CRF^ neurons.

Alcohol withdrawal, which is known to cause increased anxiety-like behavior [7], has been shown to increase excitability of a subpopulation of putative local CRF-expressing neurons in the BNST [8]. Hu et al. reported that chronic variable mild stress (CVMS) induced anxiety- and depression-like behaviors and increased neuronal excitability of ovBNST^CRF^ neurons and that intra-ovBNST injection of R121919, a CRFR1-selective antagonist, ameliorated the CVMS-induced anxiety- and depression-like behaviors [9]. These findings suggest that enhanced neuronal excitability of ovBNST^CRF^ neurons induces anxiety- and depression-like behaviors under the pathological conditions. Hu et al. also reported that increased excitability of ovBNST^CRF^ neurons was caused by potentiation of miniature excitatory postsynaptic currents and inhibition of M-currents [9]. A similar mechanism may be involved in the enhanced excitability of ovBNST^CRF^ neurons during neuropathic pain. In addition to the BNST-intrinsic neurons, CRF-expressing central amygdala (CeA) neurons send their axons to the BNST. Asok et al. reported that optogenetic inhibition of a CRF pathway from the CeA to the BNST disrupted sustained fear [10]. Furthermore, Rouwette et al. [11] and our group [2] demonstrated that CRF mRNA expression was elevated both in the BNST and CeA of neuropathic pain model rats. These findings suggest the involvement of not only BNST-intrinsic but also CeA-derived CRF nerve terminals in the enhanced CRF signaling within the BNST during neuropathic pain.

The results of this study, together with our previous studies showing that enhanced CRF signaling in the BNST caused the aversive responses in acute pain [1] and suppressed the brain reward system in chronic pain [2], suggest that chronic pain induces negative emotional states by increasing neuronal excitability of ovBNST^CRF^ neurons.

### Electronic supplementary material

Below is the link to the electronic supplementary material.

Supplementary Material 1

## Data Availability

The datasets used and/or analyzed during the current study are available from the corresponding author on reasonable request.

## Abbreviations

BNST: Bed nucleus of the stria terminalis
CeA: Central amygdala
CRF: Corticotropin-releasing factor
CVMS: Chronic variable mild stress
ovBNST: Oval part of the bed nucleus of the stria terminalis
RMP: Resting membrane potential
SNI: Spared nerve injury
