# Supplementary material for: Chronic pain enhances excitability of corticotropin-releasing factor-expressing neurons in the oval part of the bed nucleus of the stria terminalis
Source: Mol Brain. 2024 May 3;17:22. doi: 10.1186/s13041-024-01094-6 (PMC11071157; doi:10.1186/s13041-024-01094-6)
Supplement: Supplementary file 1 — Supplementary Material 1 [file 13041_2024_1094_MOESM1_ESM.docx]

**Additional information**

Detailed materials and methods

**Animals**

All experiments were carried out in accordance with Nagoya University Regulations on Animal Care and Use in Research. All experiments were approved by the Institutional Animal Care and Use Committees of the Research Institute of Environmental Medicine, Nagoya University, Japan (approval #19232 and #19268). All efforts were made to reduce the number of animals used and to minimize the pain and suffering of animals. CRF-Cre mice (C57BL/6N-Crh<tm2(cre)Ksak>) [1] and Rosa26-LSL-tdTomato mice (Ai14: Strain #007908, the Jackson Laboratory) on C57BL/6J background were used. Animals were maintained on a 12-h light–dark cycle under ad libitum feeding and drinking conditions. Room temperature was maintained at 23 ± 2 °C. Immunohistological analysis using an antibody for PKCδ which specifically localize in the oval part within the BNST [2], was conducted to confirm the localization of CRF-expressing neurons in the ovBNST. Rabbit anti-PKCδ antibody (1:1000 ; Abcam) was visualized by Goat anti-rabbit IgG labeled with Alexa Fluor™ Plus (1:1000 ; Invitrogen).

**Surgery for the chronic pain model**

The spared nerve injury (SNI) model was used as an animal model of chronic pain [3]. Mice (8 to 12 weeks old) were anesthetized using isoflurane (induction, 3.0%; maintenance, 2.0%). An incision was made on the left thigh, and the underlying muscles were separated via blunt resection to expose the three branches of the sciatic nerve bundle. The tibial and the common peroneal nerves were tightly ligated using 5-0 silk sutures and then completely transected distal to the ligation sites, leaving the sural nerve intact. Lidocaine was topically applied to the incision site. The overlying muscle and skin were sutured in layers. Postoperatively, 0.1% gentamicin ointment was applied topically, and the mice were kept in a warming chamber for 30 min. Sham surgery included exposure of the sciatic nerve, but the tibial and common peroneal nerves were not injured. Postoperatively, the mice were housed individually. Animals that exhibited postoperative motor dysfunction were not used in later experiments. The von Frey test was performed to assess tactile allodynia. The mice were placed on the elevated wire grids for at least 30 min for habituation before the test. A series of von Frey monofilaments calibrated at 0.02, 0.04, 0.07, 0.16, 0.40, 0.60, 1.00, and 2.00 g (North Coast Medical, Inc., Morgan Hill, CA, USA) were used to stimulate the plantar surface of the hind paw. The stimulation started with the 0.16-g filament. When positive (showing escape behaviors) or negative (showing no escape behaviors) responses were observed, lower or higher weight filaments, respectively, were used in sequence. The filament weight when the responses first became negative or positive, respectively, was assigned as the mechanical threshold. In cases where continuous positive or negative responses were observed to the exhaustion of the stimulus set, values of 0.02 g and 2.00 g were assigned, respectively. The criteria for mice used in the electrophysiology experiments were that the SNI group had a mechanical threshold of 0.02 g at 4 weeks and the sham-operated group had a mechanical threshold of 0.6 g or higher at 4 weeks. A total of 16 mice, 9 in the SNI group and 7 in the sham-operated group, were underwent surgery, but one mouse in the SNI group did not meet the mechanical threshold criteria and was excluded from further experiments.

**Electrophysiology**

Animals were anesthetized with isoflurane. After decapitation, the brain was quickly transferred to the frozen cutting solution (containing, in mM, 15 KCl, 3.3 MgCl2, 110 K-gluconate, 0.05 EGTA, 5 HEPES, 25 glucose, 26.2 NaHCO_3_ and 0.0015 (±)-3-(2-carboxypiperazin-4-yl)propyl-1-phosphonic acid) oxygenated with 95% O_2_/5% CO_2_. Coronal slices (250 µm-thick) containing the BNST were prepared in ice-cold cutting solution with a vibratome (VT1200S; Leica Microsystems GmbH, Wetzlar, Germany), and incubated in artificial cerebrospinal fluid (aCSF, containing, in mM, 124 NaCl, 3 KCl, 2 MgCl_2_, 2 CaCl_2_, 1.23 NaH_2_PO_4_, 26 NaHCO_3_, 25 glucose) oxygenated with 95% O_2_/5% CO_2_ at 35°C for at least 1 h, then at room temperature. The recording chamber was perfused with aCSF saturated with 95% O_2_/5% CO_2_ at 1 ml/min at room temperature. Glass pipettes were pulled from thin-walled borosilicate glass capillaries (GC150-10; Harvard Apparatus, MA, USA) with a micropipette puller (Model P-1000; Sutter Instrument, Novato, CA, USA). The pipette was loaded with K-gluconate-based pipette solution (in mM, 138 K-gluconate, 8 NaCl, 10 HEPES, 0.2 EGTA-Na_3_, 2 Mg-ATP, and 0.5 Na_2_-GTP, pH 7.3 with KOH). The tip resistance was between 3-7 MΩ. Light illumination was delivered from a light source (470 nm, 3.1 mW/mm^2^ at maximum, Niji, Blue Box Optics).

The procedure to analyze intrinsic physiological properties of CRF-expressing neurons was as follows: i) Resting membrane potential was measured in a current-clamp mode without current injection for 60 s; ii) To assess the current-voltage (I-V) relationship, membrane current was measured while the membrane potential was first set to -60 mV, then hyperpolarized to –130 mV, and depolarized to –40 mV in 10 mV steps. iii) membrane resistance was measured by injecting −20 pA step current (500-ms duration) from 0 to −180 pA with the membrane potential held at −80 mV; iv) tau was measured by injecting −20 pA ten times with the membrane potential held at −60 mV. v) rheobase, action potential threshold, and firing rate were measured by injecting +10 pA step current (500-ms duration) with the membrane potential initially held at −80 mV. The action potential threshold was defined as the membrane potential at which the derivative of the voltage (dV/dt) exceeded 10 mV/ms. All data were acquired using a Multiclamp 700B amplifier and analyzed using the pClamp10 software (Molecular Devices, Sunnyvale, CA, USA). Data from the neurons with resting membrane potential more than −50 mV, with access resistance greater than 30 MΩ, or those in which the action potential did not overshoot were excluded from the statistical analyses.

**Statistical analyses**

Data indicate means ± SEM. Statistical analyses were conducted using GraphPad Prism (GraphPad Software Inc., La Jolla, CA, USA). Two-tailed unpaired *t* test was used for comparisons between two groups. Two-way repeated ANOVA was used to analyze the data of I-V curve and firing rate. Differences with *P* < 0.05 were considered significant.

References

1. Itoi K, Talukder AH, Fuse T, Kaneko T, Ozawa R, Sato T, Sugaya T, Uchida K, Yamazaki M, Abe M, Natsume R, Sakimura K. Visualization of Corticotropin-Releasing Factor Neurons by Fluorescent Proteins in the Mouse Brain and Characterization of Labeled Neurons in the Paraventricular Nucleus of the Hypothalamus. Endocrinology. 2014;155:4054–60. DOI: 10.1210/en.2014-1182.
2. Ueda S, Hosokawa M, Arikawa K, Takahashi K, Fujiwara M, Kakita M, Fukada T, Koyama H, Horigane SI, Itoi K, Kakeyama M, Matsunaga H, Takeyama H, Bito H, Takemoto-Kimura S. Distinctive regulation of emotional behaviors and fear-related gene expression responses in two extended amygdala subnuclei with similar molecular profiles. Front Mol Neurosci. 2021;14:741895. Doi: 10.3389/fnmol.2021.741895.
3. Decosterd I, Woolf CJ. Spared nerve injury: An animal model of persistent peripheral neuropathic pain. Pain. 2000;87:149–58. DOI: 10.1016/S0304-3959(00)00276-1.
